# Cognitive performance in children and adolescents with primary hypertension and the role of body mass

**DOI:** 10.3389/fped.2024.1369690

**Published:** 2024-04-30

**Authors:** Karolis Azukaitis, Kristijonas Puteikis, Odeta Kinciniene, Dovile Mikucionyte, Ruta Mameniskiene, Augustina Jankauskiene

**Affiliations:** ^1^ Vilnius University Hospital Santaros Klinikos, Vilnius, Lithuania; ^2^ Faculty of Medicine, Institute of Clinical Medicine, Vilnius University, Vilnius, Lithuania

**Keywords:** children, adolescents, primary hypertension, body mass index, executive functions, cognition

## Abstract

**Objective:**

Primary hypertension has been shown to affect cognitive functions in adults but evidence in the pediatric population remain scarce and equivocal. We aimed to compare cognitive functioning between children diagnosed with primary hypertension and normotensive controls, with a focus on the role of different blood pressure (BP) parameters and body mass.

**Methods:**

We conducted a single-center, prospective, cross-sectional study of children and adolescents (6–17 years old) with primary hypertension and age- and sex-matched normotensive controls. All participants underwent office BP, ambulatory BP monitoring (ABPM), and central BP measurements using an oscillometric device. Neurocognitive assessment consisted of evaluation of (i) intelligence quotient (IQ), (ii) categorical and phonemic fluency, (iii) verbal memory (verbal-logical story recall), and (iv) non-verbal computerized cognitive assessment.

**Results:**

The study included a total of 59 patients with primary hypertension (14 ± 3 years) and 37 normotensive controls (14 ± 3 years). Participants in the primary hypertension group had a significantly higher body mass index z-score (BMIz: 2.1 ± 1.4 vs. 0.7 ± 0.9, *p* < 0.001), and 85% received antihypertensive therapy. Participants with primary hypertension showed worse performance in the domains of reaction speed, attention and processing speed, visual memory, new learning, and phonemic fluency. After adjusting for BMIz, only the differences in the reaction speed tasks remained significant. None of the BP parameters was associated with cognitive outcomes after adjustment for age, sex, and BMIz. BMIz associated with tasks of visual memory, new learning, spatial planning, and working memory, independent of age and sex.

**Conclusion:**

Children and adolescents diagnosed with primary hypertension exhibit worse performance in the cognitive domains of reaction speed, attention, processing speed, visual memory, and new learning. These differences to healthy controls can be partially attributed to accompanying increase of body mass.

## 1 Introduction

The rising prevalence of primary hypertension in children and adolescents is recognized as an important global health concern. The prevalence is around four percent in those aged six years but becomes particularly high in the obese population, where almost one in six adolescents is hypertensive (1). The significance of early life blood pressure (BP) elevation is well reflected by studies reporting that childhood BP tracks into adulthood and is associated with cardiovascular outcomes, including hypertension-mediated organ damage (HMOD) (2–4). The latter is already apparent in childhood, including changes in left ventricular and vascular structure and function (5–7). Moreover, evidence suggests that pediatric primary hypertension is not a condition characterized by elevated BP alone but represents a phenotype accompanied by changes in body composition, metabolic abnormalities, immune-inflammatory alterations and increased sympathetic activity (8). Thus, the effects of primary hypertension diagnosis on other organ systems can extend beyond the isolated effects of elevated BP, as previously shown in studies of vascular alterations (5).

Brain has been described as an important target of HMOD in the adult population with relatively well-defined pathophysiologic mechanisms, including but not limited to cerebral ischemia, (micro)hemorrhages, atrophy, microvascular rarefaction and endothelial dysfunction (9). However, the evidence in the pediatric population still remains scarce (10). Exposure to elevated BP in the susceptible brain microvasculature can cause various (micro)structural injuries, particularly in the prefrontal cortex, which may affect cognitive functioning, but this remains relatively understudied (10). This may be particularly important during childhood, a critical period for cognitive development (11). The latter represents a complex and dynamic phenomenon that is also strongly influenced by socio-demographic determinants, including parental education, socioeconomic status, parenting practices, as well as cultural aspects (11, 12).

A recent systematic review indicated that the association of primary hypertension with various cognitive functioning domain deficits is already present in childhood, but the evidence is heterogenous (10). Prior studies were frequently based on indirect (proxy-reported) measurement of cognitive performance (13–17) that may not reflect subtle dysfunction. Notably, none of the studies employed a comprehensive computerized cognitive assessment that would encompass wide range of cognitive domains that may be differentially affected in the state of primary hypertension (10). Finally, the role of potential hemodynamic modifiers of the effects, such as measures of central hemodynamics (central or aortic BP) that the brain is directly exposed to (18) has been only studied in one pediatric study reporting associations with indirect, proxy-reported cognitive outcomes (13). Elevated body mass and obesity that are observed in half of children and adolescents with primary hypertension (19) may have an independent effect cognitive performance in the pediatric population as previously shown in studies focusing on body mass and cognitive outcomes in childhood (20).

In the light of existing evidence and uncertainties, we performed an exploratory study focusing on the effects of real-word diagnosis of primary hypertension on a wide spectrum of cognitive performance markers in children and adolescents. The primary aim was to compare cognitive performance between children and adolescents with a diagnosis of primary hypertension and normotensive controls using a comprehensive neuropsychological testing battery. As a secondary aim, we sought to investigate the potential differential effects of different BP measures (including central BP) and body mass on various cognitive domains.

## 2 Materials and methods

### 2.1 Design and setting

We conducted a single-center, cross-sectional study of children and adolescents with primary hypertension and age- and sex-matched normotensive controls at a ratio of 2:1. The study enrolled a convenience sample of subjects from a tertiary care hospital (Vilnius University Hospital Santaros klinikos) from June 2021 to June 2023. The following inclusion criteria were used: (i) age 6–17 years and (ii) confirmed primary hypertension (except for normotensive controls). Patients with newly diagnosed primary hypertension following referral for elevated BP and those patients with a prior diagnosis of primary hypertension undergoing pharmacological therapy were included. All participants (including controls) with (i) clinical sensorimotor, neurologic or neurodevelopmental disorders (e.g., autism spectrum disorder; including the use of medications for these disorders), (ii) pre-existing structural cardiac disease and (iii) diabetes mellitus were excluded. Primary hypertension was confirmed according to the European Society of Hypertension (ESH) guidelines and all participants in the primary hypertension group underwent examinations to exclude potential secondary causes of arterial hypertension as recommended (10). Participants were invited to take part in the study during their visits in the pediatric hypertension outpatient clinic by the treating physician. After obtaining the informed consent, all participants underwent a thorough cardiovascular and neuropsychological assessment over two days as described further.

The study was conducted in accordance with the Declaration of Helsinki and approved by Vilnius Regional Bioethics Committee (approval no. 2021/5-1348-821). The participation in the study was completely voluntarily and all participants (and their parents or legal guardians) were informed that refusing to participate will not influence their care. All parents of the participants provided written informed consent to participate in the study. Assents to participate in the study were also collected in written form from adolescents aged 12 years and older by providing them with an adapted informed assent form as required by national regulations.

### 2.2 Data sources and measurements

#### 2.2.1 BMI and BP measurements

Body mass index (BMI) was determined by measuring body weight and height, and further standardized to age and sex by calculating z-scores (BMIz) using the LMS method (21) according to the World Health Organization reference data. Obesity and overweight were respectively defined as BMIz of >2 and >1, respectively (22). Office BP was measured according to ESH recommendations using an oscillometric device validated for use in children and appropriately sized cuffs (23). Office BP values were then standardized to age, sex and height according to previously published regression equations from the Fourth Report on the Diagnosis, Evaluation, and Treatment of High Blood Pressure in Children and Adolescents (24). Ambulatory blood pressure monitoring (ABPM) was performed using Spacelabs Healthcare OnTrak Intertek 315762 device. Ambulatory BP measurements were performed at intervals of 15 min during the awake period and every 30 min during sleep. The measurement was considered acceptable with at least 70% successful measurements throughout the entire 24 h period. Central systolic BP was measured using an oscillometric device (Mobil-o-Graph, IEM) that underwent validation studies in children (25, 26) and was calibrated to brachial systolic and diastolic BP as also in the reference values study (27). Both, ABPM and central systolic BP values were then standardized to age and sex by calculating z-scores using the LMS method (21) according to published reference data by Wühl et al. and Elmenhorst et al., respectively (27, 28).

#### 2.2.2 Neurocognitive assessment

Neurocognitive assessments were conducted over two days. On the initial day, tests were administered for categorical and phonemic fluency and verbal memory. Following this, a computerised cognitive assessment utilizing Computerized cognitive assessment with the Cambridge Neuropsychological Test Automated Battery (CANTAB; Cambridge Cognition, Ltd) was conducted. On the second day, participants were administered the WASI–II task battery. These assessments were performed by a licensed clinical psychologist and lasted approximately 45 min each day. Each participant was assessed individually in a peaceful and quiet room to minimise distractions. Parents were requested to wait outside until the evaluations were complete. Cognitive tasks were administered during the first half of the day, and the family were informed to ensure their children had a good night's rest and breakfast before the assessments.

Neurocognitive assessment consisted of evaluation of both verbal and non-verbal domains:

(1) Intelligence quotient (IQ) assessed using the Wechsler Abbreviated Scale of Intelligence-II (WASI-II);
(2) Categorical and phonemic fluency (the number of nouns pronounced in one minute in a particular semantic category or starting in the same letter);
(3) Verbal memory [recall of a short 24-item verbal-logical story recall (VLS) immediately after reading, after 30 min and after 24 h];
(4) This part was dedicated to evaluate the following domains (a detailed description of each test and associated variables is presented in Supplementary Table S1; dependent variables for each of the domains are provided in parentheses):
  (a) Attention and processing speed (Match to Sample Visual Search, MTS: MTSPS82, MTSRCAMD);
  (b) Visual memory and new learning (Paired Associates Learning, PAL: PALFAMS28, PALTEA28);
  (c) Motor and mental response speed, reaction time, response accuracy and impulsivity (Reaction Time Task, RTI: RTIFMDMT, RTIFMDRT, RTISMDMT, RTISMDRT);
  (d) Sustained attention (Rapid Visual Information Processing, RVP: RVPA, RVPMDL, RVPPFA);
  (e) Spatial planning and working memory (Stockings of Cambridge, SOC: SOCITMD5, SOCMNM5, SOCPSMMT, SOCSTMD5);
  (f) Working memory capacity (Spatial Span, SSP: SSPFSL);
  (g) Working memory and strategy use (Spatial Working Memory, SWM: SWMBE4, SWMBE468, SWMBE6, SWMBE8, SWMS).

### 2.3 Statistical analysis

Kolmogorov–Smirnov test and Shapiro–Wilk tests were used to assess the normality of continuous data. Continuous data were described as means (standard deviations) or medians (interquartile range) depending on the normality of distribution. Categorical data were described as frequencies. Group comparisons of continuous data were performed by employing parametric (Student's *t*-test) or non-parametric (Mann–Whitney *U*) tests, whereas proportions were compared using the *χ*^2^ test. Correlations between two continuous variables were estimated using Spearman's rank correlation coefficients. Multivariable linear models were built to test the associations between exposure and outcomes, and to adjust for potential confounders. The independence of the association between the group variable (primary hypertension or control group) and cognitive variables was assessed through analysis of (co)variance [AN(C)OVA] models. The latter was also used to differentiate between different group [(i) primary hypertension and normotensive controls or (ii) normal weight and overweight/obese] effects on cognitive outcomes (as detailed in the Methods section).

A two-sided *p*-value of <0.05 was considered significant. Statistical analyses were performed using IBM SPSS v26.

## 3 Results

### 3.1 Participant characteristics

The study included a total of 59 patients with primary hypertension (14 ± 3 years, 71% boys) and 37 normotensive controls (14 ± 3 years, 65% boys). The groups were comparable in terms of age (*p* = 0.96) and sex (*p* = 0.5). Patients with primary hypertension had higher BMIz (2.1 ± 1.4 vs. 0.7 ± 0.9, *p* < 0.001) and were more frequently obese than the control group (58% vs. 5%, *p* < 0.001). The primary hypertension group also had higher office systolic BP z-score (SBPz), higher 24-h, daytime and nighttime BPz, and central SBPz. Fifty (85% percent) patients in the primary hypertension group were taking antihypertensive medication with the majority receiving a single agent. A more detailed description of the study population is presented in Table 1.

**Table 1:** Participant characteristics.

Characteristic	Primary hypertension (*n* = 59)	Controls (*n* = 37)	*p*-value
Age, years	14 ± 3	14 ± 3	0.96
Sex, *n* (%) male	42 (71%)	24 (65%)	0.5
BMI, z-score	2.1 ± 1.5	0.7 ± 0.9	<0.001
Obese, *n* (%)	33 (58%)	2 (5%)	<0.001
Overweight, *n* (%)	12 (21%)	9 (24%)	0.710
Systolic BP, z-score	1.7 ± 1	0.9 ± 0.9	<0.001
Diastolic BP, z-score	0.9 ± 0.8	0.7 ± 0.7	0.4
24-h systolic BP, z-score	1.1 (0.3–1.7)	0.02 (−1–0.4)	<0.001
24-h diastolic BP, z-score	0.1 ± 1.3	−0.6 ± 1	0.007
24-h mean arterial BP, z-score	0.7 (0.4–1.7)	0.2 (−0.9–0.4)	<0.001
Daytime systolic BP, z-score	0.2 ± 1.1	−0.4 ± 1.3	<0.001
Daytime diastolic BP, z-score	−0.2 (−0.8–0.2)	−0.8 (−1.6–−0.2)	0.003
Daytime mean arterial BP, z-score	0.3 (0–1.2)	−0.1 (−0.9–0.3)	<0.001
Nighttime systolic BP, z-score	1.2 (0.4–1.8)	0.3 (−0.6–0.8)	<0.001
Nighttime diastolic BP, z-score	0.6 (−0.3–1.1)	−0.2 (−0.8–0.6)	0.001
Nighttime mean arterial BP, z-score	1.3 (0.7–2)	0.2 (−0.4–0.9)	<0.001
Systolic BP dipping, %	10 ± 5.1	9.4 ± 6.7	0.7
Diastolic BP dipping, %	16 (11–21)	16 (12–20)	0.99
Central BP, z-score	1.2 ± 1.9	0.2 ± 1.2	0.01
Antihypertensive therapy, *n* (%)	50 (85)	0	–
1	45 (76)	0	–
2	3 (5)	0	–
3	2 (3)	0	–

Data presented as mean ± SD or median (IQR), as appropriate.

BMI, body mass index; BP, blood pressure.

### 3.2 Cognitive performance comparison between primary hypertension and control groups

**Figure 1:**
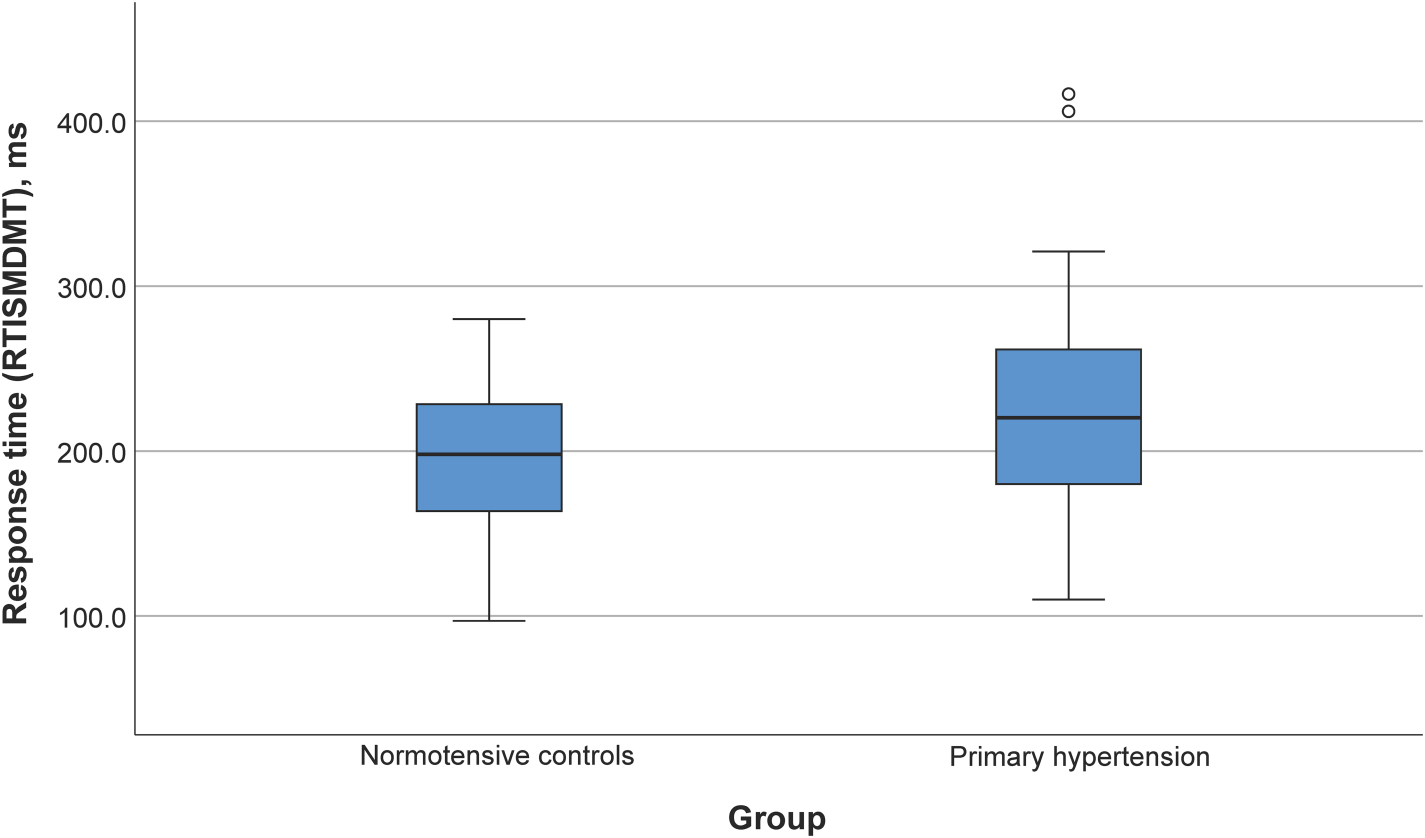
Reaction time (RTISMDMT) comparison between primary hypertension and control groups.

**Figure 2:**
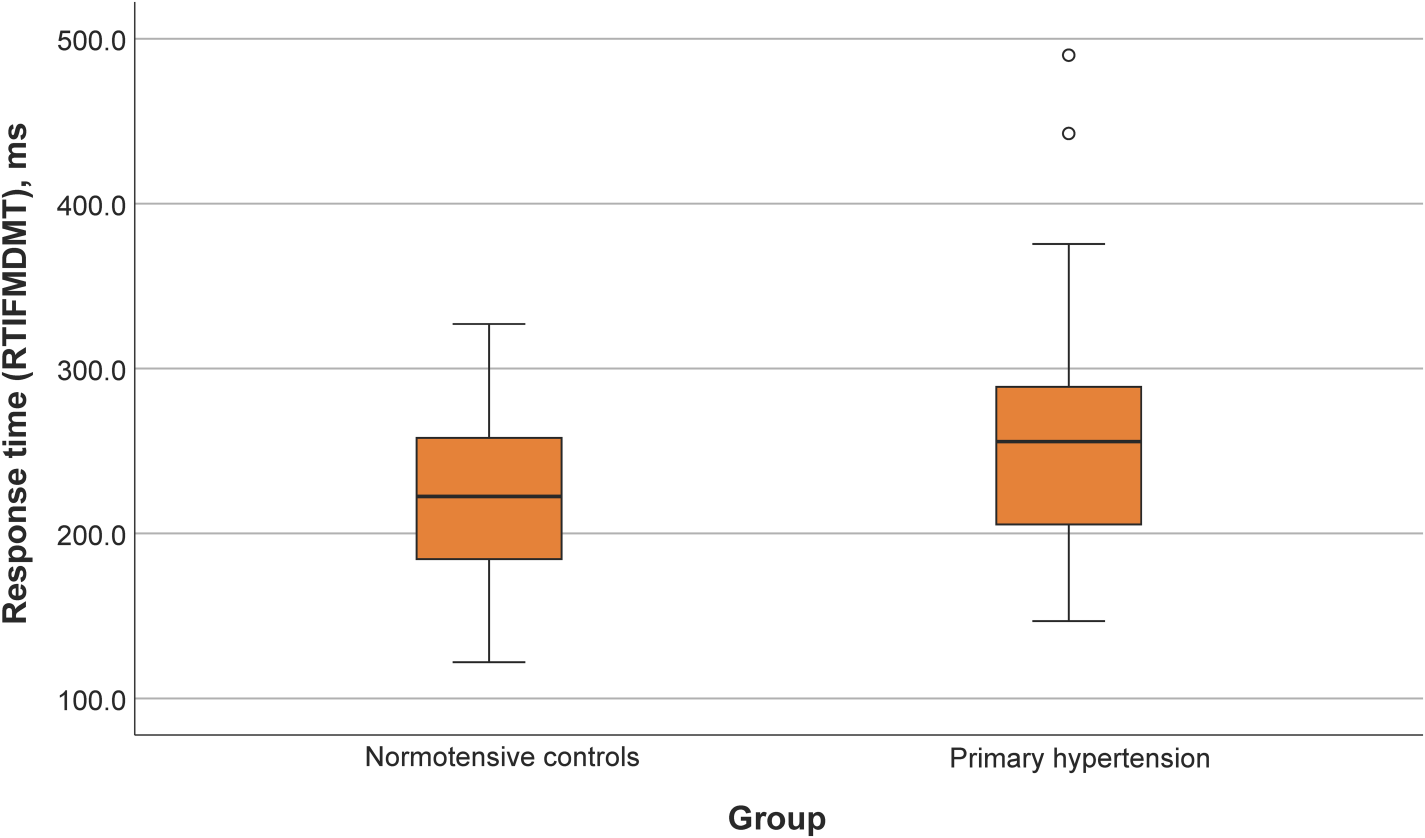
Reaction time (RTIFMDMT) comparison between primary hypertension and control groups.

Participants with primary hypertension showed worse performance in several domains of the CANTAB tasks, including reaction time (RTISMDMT and RTIFMDMT), attention and processing speed (MTSRCAMD), and visual memory and new learning (PALTEA28 and PALFAMS28). Additionally, worse phonemic fluency was observed. After adjustment of between-group differences for BMIz, only the differences for reaction speed tasks (RTISMDMT and RTIFMDMT) remained significant (*p* = 0.03 and *p* = 0.009, respectively) (Figures 1, 2). There were no differences in categorical fluency, verbal memory, IQ values or other CANTAB tasks (all *p* > 0.05). A detailed comparison of all cognitive measures between the primary hypertension and control groups is provided in Table 2.

**Table 2:** Comparison of neurocognitive performance results between primary hypertension and control groups.

	Primary hypertension (*n* = 59)	Controls (*n* = 37)	*p*-value
Verbal fluency			
Categorical fluency, units^↑^	17.6 ± 6.3	18.4 ± 6.4	0.6
Phonemic fluency, units^↑^	5 (3–8)	6 (5–10)	0.04
Verbal memory			
VLS immediate, points^↑^	12.2 ± 4.3	12.5 ± 3.7	0.7
VLS 30 min, points^↑^	10.4 ± 4.5	10.6 ± 3.7	0.8
VLS 24 h, points^↑^	10.1 ± 4.3	10.4 ± 3.6	0.7
Intelligence			
Verbal IQ, points^↑^	104 (94–114)	109 (104–115)	0.2
Non-verbal, IQ, points^↑^	102 (94–113)	107 (97–118)	0.2
IQ, points^↑^	106 (95–116)	113 (110–116)	0.08
Attention and processing speed			
MTSPS82, ms^c^	2,042 (1,622–2,425)	1,824 (1,443–2,268)	0.1
MTSRCAMD, ms^↓^	2,133 (1,809–2,490)	1,809 (1,542–1,998)	0.01
Visual memory and new learning			
PALFAMS28, points^↑^	15 (12–18)	17 (16–19)	0.02
PALTEA28, points^↓^	6 (2–12)	3 (2–5)	0.01
Reaction speed			
RTIFMDMT, ms^↓^	256 ± 66	221 ± 48	0.007
RTIFMDRT, ms^↓^	338 (322–373)	338 (318–361)	0.5
RTISMDMT, ms^↓^	224 ± 62	197 ± 44	0.02
RTISMDRT, ms^↓^	311 (288–328)	304 (289–319)	0.2
Sustained attention			
RVPA, arb. units^↑^	0.8 (0.8–0.9)	0.9 (0.8–0.9)	0.1
RVPMDL, ms^↓^	436 (402–511)	442 (394–496)	0.6
RVPPFA, arb. units^↓^	0.01 (0–0.02)	0.01 (0–0.01)	0.9
Spatial planning and working memory			
SOCITMD5, ms^↓^	7,009 (4,602–14,234)	6,898 (3,463–10,029)	0.191
SOCMNM5, points^↓^	6.5 (5.4–7.1)	6 (5.1–7)	0.3
SOCPSMMT, points^↑^	9 (7–10)	9 (8–11)	0.7
SOCSTMD5, ms^↓^	344 (0–1,180)	231 (0–904)	0.6
Working memory			
SSPFSL, points^↑^	7 (6–8)	7 (6–9)	0.4
Working memory and strategy use			
SWMBE4, points^↓^	0 (0–0)	0 (0–0)	0.6
SWMBE468, points^↓^	6 (0–12)	4 (0–12)	0.4
SWMBE6, points^↓^	0 (0–3)	0 (0–1)	0.06
SWMBE8, points^↓^	5 (0–9.5)	4 (0–11.5)	0.7
SWMS, arb. units^↓^	8 (6–9)	7 (3–8)	0.06

Data presented as mean ± SD or median (IQR), as appropriate.

ms, miliseconds; IQ, intellect quotient; VLS, verbal logical story recall.

^↑^ indicates variables where higher value represents better performance, ^↓^ indicates worse performance, while ^c^ indicates value with complex interpretation. Detailed description of CANTAB tasks is provided in Supplementary Table S1.

### 3.3 Associations between different BP parameters and cognitive performance in the overall group

In the overall group, office SBPz did not correlate with any of the cognitive outcomes, while office diastolic BPz correlated with better spatial working memory results [*ρ* = 0.25, *p* < 0.02 (SWMS)]. Central SBPz correlated with worse phonemic fluency (*ρ* = −0.25; *p* = 0.03), worse verbal memory (VLS at all three time points: *ρ* = −0.28, *ρ* = −0.27 and *ρ* = −0.29; *p* = 0.01, *p* = 0.02 and *p* = 0.01, respectively), worse sustained attention [*ρ* = −0.23, *p* = 0.04 (RVPA)], better spatial planning and working memory tasks [*ρ* = −0.24, *p* = 0.04 (SOCITMD5)] and worse working memory capacity [*ρ* = −0.30, *p* = 0.007 (SSPFSL)]. Of the 24-h ABPM parameters, 24-h SBPz correlated with better verbal memory (VLS at 24-h: *r* = 0.23, *p* = 0.04), while 24-h diastolic BPz correlated with better sustained attention [*r* = −0.25, *p* = 0.02 (RVPPFA)] and 24-h mean arterial BPz also correlated with better sustained attention [*r* = −0.213, *p* = 0.01 (RVPA)]. None of the associations remained after adjusting for age, sex and BMIz in multiple linear regression models.

### 3.4 Associations between BMIz and cognitive performance in the overall group

Higher BMIz correlated with worse phonemic fluency (*ρ* = −0.22, *p* = 0.03), worse visual memory and new learning [*ρ* = −0.28, *p* = 0.008 (PALFAMS28), *ρ* = 0.23, *p* = 0.03 (PALTEA28)] and worse performance in spatial planning and working memory tasks [*ρ* = 0.28, *p* = 0.007 (SOCMNM5), *ρ* = −0.22, *p* = 0.04 (SOCPSMMT), *ρ* = 0.27, *p* = 0.01 (SOCSTMD5)]. After adjusting for age and sex in linear regression models, this relationship remained statistically significant for visual memory and new learning [*β* = −0.28, *p* = 0.008 (PALFAMS28)], spatial planning and working memory [*β* = 0.29, *p* = 0.007 (SOCMNM5), *β* = −0.22, *p* = 0.03 (SOCPSMMT)], but not for other measures (phonemic fluency, PALTEA28 and SOCSTMD5; all *p* > 0.05).

The main effect of belonging to the overweight or obese group, but not the primary hypertension/control group, was statistically significant in the univariable ANOVA with PALFAMS28 (measure of visual memory and new learning) as the dependent variable (*p* = 0.02). The effect of the primary hypertension/control group was statistically significant in analogical ANOVA models with reaction time measures as dependent variables [*p* = 0.04 (RTISMDMT) and *p* = 0.008 (RTIFMDMT)]. No significant group (normal weight/obese or overweight) by group (primary hypertension/control) interactions were detected in the latter models.

## 4 Discussion

In the present study we explored cognitive performance of children and adolescents diagnosed with primary hypertension and normotensive controls using a comprehensive neuropsychological test battery. We hypothesized that children and adolescents with primary hypertension may exhibit subtle differential impairments in cognitive performance measures that relate to the diagnosis of primary hypertension and due to its complexity to other associated alterations (i.e., elevated body mass). Compared to age- and sex-matched normotensive peers, children and adolescents with primary hypertension exhibited worse performance in computerized tasks addressing reaction time, attention and processing speed, visual memory and new learning, and phonemic fluency. However, only differences in reaction time remained significant after adjusting for BMI, suggesting it as an important potential effect modifier. Indeed, BMI itself associated with the tasks of visual memory and new learning, while none of the cognitive domains were associated with parameters of BP. Notably, between-group differences were evident irrespective of the majority of participants in the primary hypertension group receiving antihypertensive therapy.

Several prior studies have reported differences in relatively wide-range of neurocognitive domains in children and adolescents with primary hypertension compared with normotensive controls (10). The negative effects of elevated BP on the cognition in children and adolescents are further emphasized by the data from the Young Finns Study that demonstrated associations between childhood-onset BP elevation with cognitive functioning in mid-life (29). A recent systematic review on neurocognition in pediatric primary hypertension highlighted the scarcity of studies compared to adult population. The authors also noted gaps in the existing literature, including the heterogeneity of findings, lack of studies employing direct assessment of neurocognitive functions, role of potential confounders and lack of studies assessing the effects of treatment or central hemodynamics (10).

Previous studies in the field have reported deficits in short-term and working memory, attention, fine motor dexterity and verbal fluency (30, 31). In addition, studies employing indirect (proxy-reported) evaluations have reported potential disparities in cognitive functioning and a higher prevalence of internalizing behaviors (17). In our study, we found evidence for potential deficits in visual memory and new learning, attention, processing speed and phonemic fluency among children and adolescents diagnosed with primary hypertension. These findings are in line with those of prior studies and strengthen the evidence for domain-specific injury (10). On the other hand, we identified differences in the tasks assessing reaction time, a finding that has not been previously shown in pediatric studies, but has been observed in the elderly hypertensive population (32). It is important to note that proxy-reported rating scales (such as previously applied parental questionnaires) provide a general assessment of everyday functioning, as assessed mostly through observation of behavior in a real-life setting. Whereas, direct performance assessments as employed in our study may be more accurate in detecting subtle changes in a structured environment (33) that may be more important in the context of understanding the effects of primary hypertension in childhood and more relevant for HMOD assessment.

One of the major findings of our study was the apparent effect of BMI on cognitive performance in children and adolescents with primary hypertension. This is important as elevated BP in childhood primary hypertension is frequently accompanied by increased BMI (19, 34). In our study, the prevalence of obesity (58 percent) in the primary hypertension group was comparable to that reported in prior studies in referral settings (19) and 10-fold higher than that in normotensive controls. BMI in our study population showed a negative association with visual memory and new learning, and spatial planning and working memory. Higher BMI in childhood has been linked to worse executive functions and prior studies in the pediatric primary hypertension population have suggested that BMI might be a mediating factor for cognitive dysfunction, but both studies used indirect (parental) assessments (15, 17). Collectively, this suggests that worse cognitive performance in children and adults with primary hypertension is at least in part determined by the increased BMI. Notably, the effects of BMI may be itself mediated by the associations with sleep apnea and disordered breathing (35, 36) and empirical evidence suggests negative associations of disturbed sleep with cognitive performance (37). Several longitudinal and cross-sectional studies revealed that externalising (38–40) and internalizing (38, 39) difficulties could also be related to lower task-based cognitive performance in community-dwelling children and adolescents. Thus, it is important to acknowledge that correlates of psycho-emotional wellbeing that have been out of the scope of our study may have had a mediating effect on cognitive performance.

Finally, we analyzed different measures of BP, including office BP, ABPM and central BP but were unable to find associations with cognitive performance domains after adjusting for age and sex. Dose-dependent association of BP level and cognitive functions has been reported in populational studies (30) and the hypertensive population (14, 15, 41) and it has been shown that ABPM may be superior to office BP in discriminating those with worse cognitive performance (15, 41). Considering the direct exposure of brain circulation to central BP and known disparities between brachial and central BP, particularly in young subjects, central hemodynamics could be a superior risk factor for cognitive dysfunction (18). Central BP has only been addressed in one study of children with primary hypertension and hypertension secondary to kidney disease which used a proxy-reported cognitive assessment and reported that poorer cognitive functioning was associated with higher central BP (13).

Our study population well represents the previously reported phenotype of pediatric primary hypertension, i.e., predominantly consisting of adolescents, higher frequency of males and high prevalence of obesity (1, 8, 19). However, it can also be noted to include a high proportion of participants with antihypertensive therapy that relates to the tertiary care and referral setting of our center where patients with more severe hypertension are typically followed. Although BP values among participants with primary hypertension were still higher than normotensive controls, the use of antihypertensive medications may still have blunted the associations between the BP parameters and cognitive performance. It is nevertheless worth noting that cognitive function remains worse even among children and adolescents on antihypertensive treatment compared to normotensive peers. Although it is difficult to infer the effects of antihypertensive therapy on cognitive performance due to exploratory nature of this study, these findings may also suggest that the observed differences are not solely determined by BP levels alone. Importantly, two previous longitudinal studies have reported conflicting results on the effects of antihypertensive treatment. While one study demonstrated improvements in parent-reported cognitive functioning, another study directly assessing performance showed no significant improvement after 12 months of antihypertensive treatment compared with normotensive controls (42, 43).

Our study is subject to several limitations. First, the majority of the children and adolescents in the primary hypertension group were receiving antihypertensive medications, which may have attenuated the potential associations between BP parameters and cognitive outcomes. We were unable to address this issue by performing subgroup analyses due to the low number of patients without antihypertensive treatment. Second, despite good matching for age and sex, the control group had a lower BMI. In addition, owing to the inherent nature of observational cross-sectional studies, it is impossible to control for unknown confounders and infer causality. It should also be acknowledged that we did not assess the symptoms of internalising (e.g., anxiety, depression) or externalising (e.g., hyperactivity, conduct disorders) difficulties, and sleep disruptions that may have had and independent effect on cognitive performance. Nevertheless, the comprehensive assessment of neurocognitive functioning, including paper and pencil tests, computerized battery and IQ testing represent important strength of our study. Finally, the present state of the tested cohorts (including normotensive controls) also better corresponds to real-life settings, and allows the derivation of the effects of primary hypertension diagnosis as opposed to elevation of BP alone.

## 5 Conclusions

Overall, our findings suggest that primary hypertension in childhood and adolescence is associated with changes in cognitive performance that can be largely attributed to increased BMI characteristics of this population but also the diagnosis of primary hypertension itself. Both of these factors appear to exert differential effects on the impairment of different cognitive functioning domains. Our results may be in line with the hypothesis that primary hypertension in childhood is not a disease of elevated BP but represents a state of neuro-immuno-metabolic dysfunction (8). Apparent deficits in cognitive performance, including reaction time, attention and processing speed, visual memory, new learning, and phonemic fluency, adds to the evidence of the burden associated with primary hypertension and obesity during childhood. The presence of those impairments during a critical period of development implies the need to include comprehensive neurocognitive outcomes in longitudinal and interventional studies that address childhood obesity and primary hypertension.

## Data Availability

The raw data supporting the conclusions of this article will be made available by the authors, without undue reservation.
